# Investigating the Enduring Impact of a Community-Based Health Education Program to Promote African American Elders’ Use of Technology Designed to Support Chronic Disease Self-Management

**DOI:** 10.3390/geriatrics3040070

**Published:** 2018-10-13

**Authors:** Charles R. Senteio

**Affiliations:** School of Communication and Information, Rutgers University, New Brunswick, NJ 08901, USA; charles.senteio@rutgers.edu; Tel.: +1-848-932-7586

**Keywords:** community health informatics, technology for self-management, community-based participatory research, racial disparities, community-based health education, elder African Americans, intergenerational technology knowledge transfer

## Abstract

Elders experience chronic disease disparities and barriers to access technology designed to support recommended self-management behaviors. Elders from racial minority groups are among those who experience particular disparities in chronic disease incidence, outcomes, and barriers to technology use. In order to investigate strategies to address barriers, the study team recruited elder African Americans with diabetes and young adults connected to the elders through naturally occurring familial or social networks. Participants attended a community-based health education session focused on enhancing self-efficacy for recommended self-management and using consumer-oriented technology accessible on their smartphones for self-management support. To assess enduring impact, the study team conducted a pilot study to investigate perceptions and use one month following the health education session. Both elders and young adults offered perspectives on what was effective in teaching elders how to use technology. Both age groups stressed that having patience was crucial, as is providing encouragement for the elders to try tasks on their own. Both elders and young adults also showed a statistically significant increase in aspirations to work together for additional intergenerational health and technology knowledge exchange. Several elder participants continued using technology that they first used during the session. This novel, pilot study describes how to promote self-management and technology use for individuals plagued by persistent chronic disease and technology use disparities.

## 1. Introduction

In the United States, individuals aged 65 or older are at increased risk of having chronic conditions when compared to younger age groups, as one third of elders have at least one of the common “Big Five” chronic conditions: diabetes mellitus, cardiovascular disease, chronic respiratory disease, cancer, and stroke [1]. Striking racial disparities persist for these common chronic conditions. When compared to White elders, African American elders are twice as likely to have diabetes and associated complications such as blindness and amputations [2].

Self-management behavior—specifically medication, diet, routinized physical activity, and attendance at follow-up appointments—represents the vast majority of chronic disease management recommendations, and individuals who are able to follow health behaviors consistent with recommended self-management experience better health outcomes than those who experience barriers that prevent them from following recommended behaviors [3,4]. Rapid advancements in mobile computing capabilities (i.e., smartphones, smartwatches, tablets) and communications (i.e., broadband, cellular networks) have resulted in a proliferation of consumer technology designed to support recommended chronic disease self-management [5]. Diabetes patients may use mobile applications to help track medication behavior, physical activity, and dietary choices [6,7]. Also, smartphone users have ever-increasing choices for apps designed to support hypertension self-management, with capabilities like telemonitoring of blood pressure [8]. Patients with cardiovascular disease may use smartphone apps to help monitor vital signs and detect abnormalities in heart functions [9]. Cancer patients may use apps as sources of health information to help answer questions regarding symptoms [10]. However, effective use of these mobile applications requires considerable digital literacy [11,12].

Both age and race are associated with barriers to using information and communication technologies (ICTs) to support self-management. Elders’ decisions to use ICTs are influenced by agency and personal preference [13,14]. Elders who are economically disadvantaged are less likely to use ICTs, due to barriers to access technology (i.e., broadband, tablet) [15] and level of health literacy, which is the capacity to access, understand, and communicate health information [16,17,18,19,20]. Despite these research insights, there remain gaps in the literature on the effectiveness—defined by increases in health literacy, and self-efficacy—of consumer-oriented technology tools used by elders with chronic conditions [21], particularly for African American chronic disease patients who are more likely to have low health literacy [22] and, as stated, are at higher risk of having chronic conditions.

### Self-Management Support via Community-Based Health Education

Although the concept of self-management, which is grounded in Bandera’s social learning theory, was developed in the 1960s and self-efficacy followed in the early 1970s [23], ‘self-management’ has emerged relatively recently as healthcare delivery systems have attempted to shift from treatment, towards health promotion and health maintenance [24]. Chronic disease self-management education programs can improve patients’ self-efficacy, knowledge, and adherence to recommended self-care [25,26,27,28,29], and program effectiveness is enhanced through use of technology, primarily by facilitating improvement in heath literacy. Findings from community-based health education initiatives describe barriers to self-management while illustrating the importance of language and culturally appropriate health information for common chronic conditions, specifically diabetes [30,31], cardiovascular disease [32], and cancer [33,34].

The community-based participatory research (CBPR) approach [35] has enabled patients to have a voice in further elucidating barriers to self-management and health information needs [36,37]. In limited studies, including community members from target populations helping to design health education activities, CBPR has been shown to further enhance program effectiveness and helps to address known barriers [38,39]. However, there is little research to help guide development and execution of effective community-institutional collaborative health promotion efforts focused on ethnic minority populations who experience disparities [40,41]. Also, studies have not investigated impact on self-efficacy for following recommended self-management behaviors in the months following a community health intervention which individuals from the target population helped to design [42]. To address these gaps in the literature, this paper describes a pilot study that examines the impact for African Americans one month after a community-based health education intervention designed to enhance self-efficacy and interest for using technology to support chronic disease self-management.

## 2. Materials and Methods

This study used a mixed method study design. The study team collected quantitative data through a paper-based survey, then conducted phone interviews that included open-ended questions, the answers to which comprised the qualitative data. The study sample was comprised of elder (aged 50 or older) African Americans with diabetes and young adult African Americans (aged 18–49) connected to the elder participants via family or naturally occurring social relationships. Participants were residents of one of the two study sites—one in Detroit and one in Flint, Michigan. All study procedures were replicated at both sites. Our purposive sampling included these two urban areas because they are particularly impacted by chronic disease racial disparities. The study demarcates “elders” as aged 50 or older. There is no established age threshold for classifying “elder” in the health promotion literature. For United States-based studies that use secondary data sets to investigate elder health and technology use (i.e., Center for Medicare and Medicaid, National Health and Aging Trends Study—NHATS), 65 is a common age threshold [43,44]; however, 50 is a common threshold for studies similar to this one that investigate technology use and use primary data [45].

The Rutgers University Institutional Review Board (IRB) approved the study protocol on 4 July 2016 (IRB #: 16-793). Participants were recruited through relationships with community members residing in two urban centers in Michigan. These relationships were established and nurtured through an established academic–community partnership between the study team and a faith-based organization with a history of health promotion activities in both sites. The study team worked closely with staff to identify and screen potential participants. Inclusion criteria for elders included a self-reported diabetes diagnosis, and younger adult participants had to be connected to the elder via familiar or naturally occurring social networks. Each participant was compensated $40 for their time.

The study team collaborated with individuals selected from the target population to design the health education intervention that was informed by the socio-ecological model of health and the Senior Technology Acceptance and Adoption Model (STAM). The design process [46] and post-intervention results are detailed in other publications [47]. The three-hour, interactive health education session was designed to promote intergenerational technology transfer between elders with diabetes and younger adults connected to them. After a review of diabetes risk factors, symptoms, and treatment options, the session featured elder–young adult pairs working together for 20 min of the three-hour session. The dyads chose a specific task associated with self-management behavior (i.e., downloading a health app focused on nutrition, performing a web search for medication information or exercise information, etc.), then used their own smartphones to perform that specific task. This study utilized a self-report pre- and post-test design, and an instrument that measured self-efficacy, preference for learning about, and using technology which was administered one month after the sessions. This enabled a longer-term evaluation of the effectiveness of the intervention. The study team selected one month as a follow-up time period to administer the questionnaire in order to measure potential impact. Although 2–6 months is considered a desirable tracking time period across behavioral science studies focused on self-reported health behavioral change [29,48], the study team selected one month to maximize response rate, an important consideration given that the study population is characterized by low participation and retention in health promotion programs [49].

This study facilitated two health education sessions at each of the two study sites. Participants completed a paper-based questionnaire at the beginning of the health education sessions (referred to as *pre-session*) which included sociodemographic information (i.e., age, gender, race, health insurance status, household income, etc.), and at the conclusion of each session (referred to as *post-session*). The consent forms included the option to be contacted for the follow-up telephone interview. Participants were contacted by telephone by the study team one month following the session (referred to as *telephone interview follow-up*). Participant codes were assigned when the *pre-session* survey was completed, and the study team used the participant codes to assign responses *post-session* and at *telephone interview follow-up* to the same participant.

The telephone interview follow-up instrument included 29 total questions (please see Appendix A; Telephone Follow-up Interview Guide). The first 11 questions are validated questions to solicit self-efficacy levels using a 5-point Likert scale and they were the same for each of the three times the survey was administered (5—Strongly Agree, 4—Agree, 3—Neutral, 2—Disagree, 1—Strongly Disagree). The instrument includes questions administered only to elder participants, and other questions administered only to young adult participants. For elder participants, the questionnaire solicited preference for learning about technology and use since the health information session (#12–14); it also includes a question soliciting if the elder has used technology that they learned about during the health information session (#15). For young adult participants the instrument included questions soliciting preference for learning about health and validated questions to assess self-efficacy for teaching elders how to use technology (#16–18); it also includes a question soliciting if the young adult has worked with an elder to help them use technology since the health information session (#20). The instrument ends with nine open-ended questions concerning participants’ use of technology since the health information session.

The instrument was designed using validated assessment tools to measure health education session impact for self-efficacy concerning technology used to support diabetes self-management [50,51,52,53]. To test the impact of the intervention in enhancing the use of technology for diabetes self-management skills, responses were compared using test for difference between the:

- *pre-session* and *post-session* responses;
- *pre-session* and *telephone interview follow-up* responses;
- *post-session* and *telephone interview follow-up* responses.

Distribution of the data was tested for normality first, and based on the test result a repeated measure test (McNemar’s Chi Squared) was employed to compare the responses. The comparison test was performed for the elder and young adult groups individually, as well as for the entire sample.

The qualitative data was derived from the open-ended questions asked during the telephone interviews. Our study team performed thematic analysis. This qualitative analysis method was selected because it is appropriate for analyzing potentially variant perspectives from different participants (i.e., elders and young adults), and the method lends itself to highlighting both differences and similarities between responses [54,55]. All but three of the phone interviews were conducted by the author, and the remainder by another member of the study team (Co-PI). The Co-PI was also present during the health education sessions. We controlled for interviewer variation by comparing our notes and discussing the interview notes, each of which were recorded. A research assistant took notes during the interviews and we discussed and refined the notes following each telephone interview. The notes served as the qualitative data.

Members of the study team undertook a six-step approach to analyze the qualitative data by performing thematic analysis [56] of the answers to the open-ended questions in the instrument. First, we familiarized ourselves with the data, which included our own notes as both the author (PI) and the Co-PI had been involved in the study since the outset. Next, we generated initial codes, which emerged from answers to the open-ended questions that were designed to solicit data on specific points reflected in our research questions. In the third step, we developed our initial themes, and refined them during step four. In the fifth step, we finalized the themes, being careful to identify justification, in part, informed by our personal insights based upon this study, and previous work. The last step involved preparation of the final write up based on the completed analysis, which is reflected in the results section to follow.

## 3. Results

### 3.1. Sample

The study team helped conduct the health education sessions in the two study sites on 20 and 21 May 2017. We conducted follow-up telephone interviews between 20 June and 11 July 2017. All participants were African American and every participant contacted answered every question in the instrument. Overall, the average age of all 18 participants was 52 years (*M* = 51.56, *SD* = 17.06), although two thirds of participants were elders (12 of 18; 66.7%) (see Table 1). The youngest participant was 20 years old and the eldest was 79 years old. As individual groups, average age of elder participants was 61 years old (*M* = 61.4, SD = 6.79; min = 53, max = 79), whereas the average age of the young adult participants was 31 years old (*M* = 30.50, SD = 9.52; min = 20, max = 46). The age distribution for all participants was tested for normality and for elder and young adult groups separately by using Shapiro–Wilk’s method. The age distribution was found to be normal among the entire sample (*p* = 0.06173) and also for the elders (*p* = 0.0157) and for the young adults separately (*p* = 0.7011).

### 3.2. Impact of Intervention: Pre-Session; Post-Session; Telephone Interview Follow-Up—Elders

Elders showed statistically significant improvements for all three statements regarding their inclination to learn more about technology, including technology that can help with self-management.

1. I like learning about technology from young people;
2. I would welcome learning more about technology in general from young people;
3. I would welcome learning more from young people about technology that can help me manage my health.

Elders indicated a significant improvement, between *pre-session* and *post-session* for all three statements, and the *telephone interview follow-up* responses indicated improvements compared to *pre-session* responses (see Table 2). The test results for the elders’ group suggest an increase in use of technology and for self-efficacy for use of technology, although none of the increases are significant. Elders indicated an increase in using websites to acquire health information (*TECH3*, from mean response *pre-session* = 2.83, *post-session* = 3.33; *telephone interview follow-up* = 3.75) (see Table 2). Elders also indicated an increase in self-efficacy for being able to get the help they may need for using technology to support their health, and the increase remained at the post-session level at telephone interview follow-up (*TECH2*, from mean response *pre-session* = 3.58, *post-session* = 4.08; *telephone interview follow-up* = 4.08).

#### Three Themes That Emerged One Month after the Session—Elders

As shown in the tests of difference between the *pre-session*, *post-session*, and *telephone interview follow-up,* the elders indicated aspirations for learning more about, and actual use of, technology that they learned about at the session. Responses to the open-ended questions provide further context for elder participants’ use since the session, and reveals their perspectives. Three key themes emerged in analyzing the elders’ responses. First, elders used technology designed to support self-management that they were introduced to at the session; they used this technology by themselves and with others. Second, elders enjoy working with young adults as they learn about new technology. Last, elders shared their perspectives on what specific approaches are effective in teaching them how to use technology, and they affirmed that smartphones are an accessible, suitable technology.

Seven of the 11 elders stated that they have used specific technologies that they learned about at the session, primarily for seeking health information. One elder participant indicated that he used an app to look up information on his medication, “I learned about a program to look for certain medication. I went to look up more information about my medication. I didn’t know about that before [the session]. (My daughter) showed me how to go to Google and look up medications side effects. I typed into the phone. I was able to do that after [the session]” (Male, 69 years old). Another participant shared that he has used websites since the session, “I can got to (web) pages and do all the steps. I learned about that [at the health information session]” (Male, 63 years old). Another participant mentioned that she used social media, which she had not used prior to the session, “I learned about Facebook [at the session], by working together with young people” (Female, 62 years old). For another participant the session gave him an opportunity to be introduced to technology, “I hadn’t even used a website. And I learned how to use email, and now I can use [email]” (Male, 79 years old). One elder also recounted working with his granddaughter since the session, “Yes I went to a website … my little girl pulled up the website on the laptop. (It was) very educational” (Male, 65 years old). Another participant described how he learned how to download a smartphone app at the session, as a consequence he better understands how apps can help him find health information, “Other people showed me how to go to the store to download an app to look for medications, and what to eat” (Male, 79 years old).

Elders also shared their enjoyment in working with young adults to learn about technology, “I like working with young people to learn something new” (Female, 62 years old). Another expressed how enjoyable it was to work with his granddaughter, “Learning from younger people is good. I felt good working with my granddaughter (22 years old), she’s very bright” (Male, 79 years old). Another mentioned working with her daughter, “I have a bad memory, so I’ve worked with my daughter. She helps me when I have a problem” (Female, 53 years old). Another expressed how beneficial it was to learn from other attendees whom he knew, “I learn more from a friend than a stranger” (Male, 79 years old). He also stated how the comradeship was similar to what he experiences at church, “We learned from each other. I like that. I would enjoy another seminar, whenever it may come … doing the same thing. It’s like a prayer meeting” (Male, 79 years old). Part of the enjoyment was due to participants affirming that they were not the only ones who were in their situation, “Someone else described things that I also experienced. (It) made myself more conscious. I’m not the only one going through this” (Male, 69 years old).

Elders also expressed specific approaches that will help them learn about technology; patience was an important aspect of the approach the recommend. “Have more patience and passion. The old person may not understand. They need patience to go over and over again” (Male, 57 years old). Another stated the benefits of working with another person, “I always learn something from working with someone else” (Male, 63 years old). This participant specifically mentioned how the smartphone was a suitable technology platform for him, “I prefer to use my phone (over PCs or tablets). I’m pretty good with using my phone. Doing this kind of seminar with phones seems to work well” (Male, 57 years old).

### 3.3. Impact of Intervention: Pre-Session; Post-Session; Telephone Interview Follow-Up—Young Adults

The young adults showed improvements in all four statements associated with working with elders concerning health and technology use:

1. I like learning about health from elders.
2. I would welcome learning more about health from elders.
3. I would welcome teaching elders about using technology that can help them manage their health.
4. I feel confident showing elders how to use technology.

But young adults only indicated a significant improvement, between *pre-session* and *telephone interview follow-up* for openness for learning about health from elders (see Table 3).

#### Two Themes that Emerged One Month after the Session—Young Adults

Responses to the open-ended questions also provide further context for young adult participants’ activities and perceptions since the session. Two key themes emerged from the analysis of the responses from the young adults. First, young adults also worked with elders since the session using technology designed to support self-management. Second, young adults shared their perspectives concerning the specific approaches that work well in teaching elders how to use technology; they emphasized patience, as did the elders.

The young adults stated that they worked with other family members since the session. One participant mentioned that she has worked with the person she attended the session with, “We have been working on websites together” (Female, 46 years old). Another mentioned working with her mother since the session, “I was with my mom (at the session), and I have showed her the internet on her phone” (Female, 26 years old). Another expressed how working with elders can result in increasing the elder’s agency, and the ability for them to access information on their own, “I showed her WebMD since (the session). I showed her how to use Web MD to look up muscle pain. We sit together, working on the phone. A little more work will help her do that on her own” (Female, 35 years old). Another participant mentioned working with his partner *and* his father, who both have diabetes, “My girl and my father have diabetes, they came to me (after the session). I’ve gone on Google to help them search on the web to learn about diabetes” (Male, 33 years old).

Young adults also offered their perspectives on how best to support elders to use technology, “Young people need to be more willing to be more patient” (Female, 35 years old). Another mentioned specifics concerning patience, “Young people need to be patient, take your time. Make sure they understand before you move on” (Female, 26 years old). Being patient and engaging with elders also can confirm if the elder understands their instructions, “Be hands on with them, make sure they are following” (Male, 33 years old). Another mentioned that it is important to exude confidence when working with elders, “If you are speaking with confidence, they are adopting your confidence” (Female, 35 years old). Another mentioned promoting agency, by letting elders try to use the technology themselves, “If they are reluctant, let them know there’s only a couple of steps. Have them to do it themselves. See if they can pull it off” (Female, 26 years old).

## 4. Discussion

Study results show that while African American elders experience barriers to access technology designed to support chronic disease self-management [17], participants have a firm interest in learning about technology in general, and in learning about and using technology designed to support chronic disease self-management. They also indicate an interest in working with young adults connected to the elders via naturally occurring family and/or social networks who are willing to support them. Young adults express interest and enjoyment in learning from elders about heath. They also express confidence in showing elders how to use technology, and welcome doing so for technology designed to help with chronic disease self-management.

Both elders and young adults offered perspectives on what made this intergenerational technology transfer effective—patience and providing opportunities for the elders to perform tasks themselves were common themes across the two age groups. This finding is consistent with the literature on intergenerational knowledge transfer which states that, although elders and young adults may have disparate goals, these goals can converge around technology use [57]. Literature suggests that intergenerational technology activities focused on health can create learning opportunities for both young adults and elders. Learning is facilitated by each generational group’s desire to learn new skills, rather than centering on differences in age or technology competencies [57].

This study offers novel insights on African American elders’ interest in using technology to support diabetes self-management. Study limitations include a relatively small sample size and results are reliant on self-reported data. While the results show uptake after one month they do not evaluate sustained use, given the evaluation at one month. The study does not elucidate any changes in actual health behaviors, nor does it explore specific details regarding technology use and potential resulting increases in health information use, or subsequent health behaviors. However, given the continued proliferation of technology-enabled tools designed to support health behavior, and persistent chronic disease disparities that afflict elder and minority populations, study findings provide compelling insights regarding elders desire to learn about technology, and young adults’ desire to support them in developing the requisite knowledge to take advantage of these tools. Future research should include an examination of barriers to technology use, and the development of pragmatic approaches to address these barriers. The study session examined in this paper offers tremendous potential to scale such an intervention, as the participants brought their own smartphones with cellular network access. The elder–young adult dyads self-directed the learning and elder participants were eager to learn and do more with what they learned, and the young adults were very open to continuing to work with them. Findings offer a particularly intriguing insight into factors that promote intergenerational technology and health knowledge transfer which should be further explored in large, additional diverse populations.

## Figures and Tables

**Table 1:** Participant demographics.

	Total Sample (*n* = 18)	Elders (*n* = 12)	Young Adults (*n* = 6)
Age (years), *M* (Standard Deviation)	51.56 (17.06)	62.08 (6.79)	30.50 (9.52)
Age Range	20–79	53–79	20–46
Gender:			
Female	9	4	5
Male	9	8	1
Marital Status:			
Married	3	3	0
Single	13	7	6
Windowed	2	2	0
Divorced	0	0	0
Living Arrangement:			
Alone	12	11	1
With spouse only	0	0	0
With spouse and children	2	0	2
With children	1	0	1
Other	3	1	2
Education:			
Some high school	6	4	2
High school diploma/GED	7	5	2
Some college	5	3	2
College graduate	0	0	0
Employment status:			
Employed	4	0	4
Unemployed	2	0	2
Retired	2	2	0
Disabled	10	10	0
Household income:			
<$14,000	9	5	4
$15,000–$24,999	4	3	1
$25,000–$34,999	1	1	0
$35,000 –$49,999	0	0	0
$50,000–$69,999	1	1	0
$70,000+	0	0	0
Health Insurance:			
No	1	0	1
Yes—through employment	0	0	0
Yes—through spouse	0	0	0
Yes—Medicare	7	5	2
Yes—Medicaid	10	7	3
Yes—Other coverage	0	0	0
Difficulty in paying for healthcare treatment:			
Always	1	0	1
Very frequently	0	0	0
Occasionally	1	1	0
Rarely	2	2	0
Very rarely	2	1	1
Never	11	7	4

**Table 2:** Comparison of responses in three sessions (pre-, post-session and phone interview) for **elders** (*p* < 0.05 *, *p* ≤ 0.01 **, *p* ≤ 0.001 ***).

Survey Question	Sample	Pre-Session	Post-Session	Interview Session	Difference between the Three Sessions McNemar’s Chi-Squared Value (*p*-Value)		
		Mean response (standard deviation)			Pre v/s post	Pre v/s Interview	Post v/s Interview
I like learning about technology from young people	11	4.16 (0.57)	4.27 (0.46)	4.25 (0.85)	10 (0.04 *)	3.14 (0.200)	2.85 (0.090)
I would welcome learning more about technology in general from young people	10	4.00 (0.63)	4.27 (0.46)	4.16 (0.57)	9 (0.011 *)	3.47 (0.017)	2.80 (0.090)
I would welcome learning more from young people about technology that can help me manage my health	11	4.08 (0.66)	4.27 (0.46)	4.17 (0.57)	**10** (0.0043 **)	2.03 (0.360)	1.58 (0.201)
I have used websites to get health information. (TECH3)	12	2.83 (1.11)	3.33 (1.23)	3.75 (0.87)	6 (0.058)	5.11 (0.160)	5.18 (0.120)
I can get the help I need to use technology to help me with my health (TECH2)	12	3.58 (1.08)	4.08 (0.79)	4.08 (0.51)	6.70 (0.080)	3.40 (0.330)	3.60 (0.320)

*Note*: 5—Strongly Agree; 4—Agree; 3—Undecided; 2—Disagree; 1—Strongly Disagree.

**Table 3:** Comparison of responses in three sessions (pre-, post-session and phone interview) for the **young adults** (*p* < 0.05 *, *p* ≤ 0.01 **, *p* ≤ 0.001 ***).

Survey Question	Sample Size	Pre-Session	Post-Session	Interview Session	Difference between the Three Sessions McNemar’s Chi-Squared Value (*p*-Value)		
		Mean response (standard deviation)			Pre v/s post	Pre v/s Interview	Post v/s Interview
I like learning about health from elders	6	4.16 (0.41)	4.33 (0.52)	4.33 (0.52)	**10** (0.003 **)	4.57 (0.327)	3.81 (0.058)
I would welcome learning more about health from elders	6	4.16 (0.41)	4.33 (0.41)	4.16 (0.41)	**10** (0.021 *)	3.63 (0.017)	2.89 (0.058)
I would welcome teaching elders about using technology that can help them manage their health	6	4.16 (0.41)	4.16 (0.41)	4.67 (0.52)	**10** (0.002 **)	6 (0.42)	2.78 (0.34)
I feel confident showing elders how to use technology	6	4.16 (0.41)	4.33 (0.52)	4.33 (0.52)	2 (0.150)	2 (0.150)	0.31 (0.570)

*Note*: 5—Strongly Agree; 4—Agree; 3—Undecided; 2—Disagree; 1—Strongly Disagree.

## Appendix A. Telephone Follow-Up Interview Guide

**Sample Script:** Thanks so much for attending the session on (Flint—Saturday, 20 May; Detroit—Sunday, 21 May). At the seminar we announced that we would follow-up in about 3 weeks for a quick conversation about your current thinking about technology use. I’d like to ask you just a few questions, and I’ll be recording our conversation when I ask you the open-ended questions to wrap up. Do you have any questions before we start?

**Self Efficacy**

1. I have problems learning about my medical condition(s) because of difficulty understanding written information.
  **Strongly Agree**    **Agree    Undecided    Disagree    Strongly Disagree**
2. I feel confident when I have to fill out medical forms by myself.
  **Strongly Agree**    **Agree    Undecided    Disagree    Strongly Disagree**
3. I am confident that I can find health information that I can understand.
  **Strongly Agree**    **Agree    Undecided    Disagree    Strongly Disagree**
4. I have used a technology that I learned about at the D-Party.
  **Strongly Agree**    **Agree    Undecided    Disagree    Strongly Disagree**
5. I am confident that I can find health information that I can understand.
  **Strongly Agree**    **Agree    Undecided    Disagree    Strongly Disagree**
6. I can use technology designed to help me with my health.
  **Strongly Agree**    **Agree    Undecided    Disagree    Strongly Disagree**
7. I can get the help I need to use technology to help me with my health.
  **Strongly Agree**    **Agree    Undecided    Disagree    Strongly Disagree**
8. I have used websites to get health information.
  **Strongly Agree**    **Agree    Undecided    Disagree    Strongly Disagree**
9. I can download a health app.
  **Strongly Agree**    **Agree    Undecided    Disagree    Strongly Disagree**
10. I am confident in my ability to learn more about the available technology (e.g., mobile phone applications, electronic glucometers, websites like WebMD) which could help me with my health.
  **Strongly Agree**    **Agree    Undecided    Disagree    Strongly Disagree**
11. I am confident that I can find health information that I can understand.
  **Strongly Agree**    **Agree    Undecided    Disagree    Strongly Disagree**

**For Elder Participants**

12. I like learning about technology from young people.
  **Strongly Agree**    **Agree    Undecided    Disagree    Strongly Disagree**
13. I would welcome learning more about technology in general from young people.
  **Strongly Agree**    **Agree    Undecided    Disagree    Strongly Disagree**
14. I would welcome learning more from young people about technology that can help me manage my health.
  **Strongly Agree**    **Agree    Undecided    Disagree    Strongly Disagree**
15. I have used a technology that I learned about at the D-Party.
  **Strongly Agree**    **Agree    Undecided    Disagree    Strongly Disagree**

**For Younger Participants**

16. I like learning about health from elders.
  **Strongly Agree**    **Agree    Undecided    Disagree    Strongly Disagree**
17. I would welcome learning more about health from elders.
  **Strongly Agree**    **Agree    Undecided    Disagree    Strongly Disagree**
18. I would welcome teaching elders about using technology that can help them manage their health.
  **Strongly Agree**    **Agree    Undecided    Disagree    Strongly Disagree**
19. I feel confident showing elders how to use technology.
  **Strongly Agree**    **Agree    Undecided    Disagree    Strongly Disagree**
20. I have shown an elder how to use technology since the D-Party.
  **Strongly Agree**    **Agree    Undecided    Disagree    Strongly Disagree**

Short Answer—Please also respond to the following on your activities *since the D-Party*:

1. Have you used a technology which you learned about at the D-Party. If yes, which? If no, why?
2. Describe if you have worked with another person to help you use technology to help you manage your health.
3. Have you used the following to help you manage your health:
  - Phone app (if yes, which ones);
  - Website (if yes, which ones);
  - Other?
4. Describe how you feel about working with others to help you learn about technology designed to help you with your health.
5. Describe how your perceptions about using technology have changed since the D-Party.
6. Since we are trying to learn about how to help elders use technology to manage their health, what other things could we do to help this?
7. **For Elders**: How could younger people more effectively help you learn about technology designed to help you with your health?
8. **For Young Adults**: How could elders more effectively learn about technology designed to help them with their health?
9. Do you have anything else to share about the D-Parties, or your use of technology to help you manage your health?
